# A Reply

**Published:** 1892-07

**Authors:** 


### A REPLY.

---

In this same issue, we publish the suggestions of a brother practitioner in regard to an editorial of ours on a "Growing Evil that should be Remedied." The doctor seems to be thoroughly in accord with our views, as to the prevalence of the evil; but with the ideas of procrastination from which our profession has always suffered, simply offers palliative remedies where heroic ones should be used, or, as he says, use diplomacy and strategy, and by these subtifuges try to accomplish what only a straight out fight will.

There has never been a suggestion of reform in medicine that has not met with bitter resistance from both within and without the ranks of the medical profession; nor do the writer's suggestions tend to correct the evil, except from a monetary stand; that of causing the patient to return at stated times, by giving him a placebo that is of no benefit—this course, we think reflects upon the dignity of the profession.

It is not by a vacillating course of this kind that we can inspire confidence and respect in the minds of the laity, but by taking a firm stand when we are right, and by maintaining it in the face of all obstacles.

We do not doubt the American habit of ideal liberty, but believe that their sense of justice is just as great. That the question is after all, a matter of education; and that whenever it is presented to them properly, and they recognize that it is for their benefit, and not ours, we will gain their hearty co-operation in executing them. W. F. W.

